# Survival Analysis of Elderly Patients With Laryngeal Cancer After Total Laryngectomy: A Retrospective Cohort Study

**DOI:** 10.7759/cureus.60792

**Published:** 2024-05-21

**Authors:** Christos S Avdulla, Nicholas S Mastronikolis, Ntaniela Tachirai, Michalis Leotsinidis, Eleni Jelastopulu

**Affiliations:** 1 Department of Public Health, University of Patras, Patras, GRC; 2 Department of Otolaryngology, University of Patras, Patras, GRC; 3 Department of Public Health Policy, University of West Attica, Athens, GRC

**Keywords:** western greece, overall survival (os), tumor-node-metastasis (tnm) staging, elderly patients, total laryngectomy, laryngeal cancer

## Abstract

Objective

This study investigates the overall survival (OS) of elderly patients who underwent total laryngectomy for laryngeal cancer (LC) and examines the impact of tumor-node-metastasis (TNM) staging on survival rates.

Methods

A retrospective cohort study utilized data from the Otorhinolaryngology Clinic at the University Hospital of Patras, including 75 elderly patients (>65 years) who underwent total laryngectomy for LC between 2000 and 2015. Survival analysis was performed using the Kaplan-Meier estimator, with comparisons made using the Log-rank test. Statistical significance was defined as the p-value being less than or equal to 0.05.

Results

Over the 16-year period, new LC cases were predominantly male (97.3%) with a mean age of 73.88 years (range: 65-89 years). Most patients were smokers (96%) and alcohol users (54.7%). Histologically, 18.7% of tumors were classified as poorly differentiated, 65.3% as moderately differentiated and 16% as well differentiated. Post-surgical TNM staging indicated 10.7% stage II, 37.3% stage III and 52% stage IV, primarily located in the glottis (62.7%) and followed by supraglottis (34.7%). All patients underwent total laryngectomy, with 69.3% and 37.3% receiving neck dissection and adjuvant therapy (chemotherapy or radiotherapy), respectively. During follow-up, 39 patients died, with 74.3% due to disease-related causes. Five-year OS rates were 44.6%, with variations by stage (stage II: 62.5%, stage III: 55.8%, stage IV: 32.4%; p=0.039) and age (65-75 years: 51.7%, >75 years: 34.7%; p=0.039).

Conclusions

TNM staging of the laryngeal cancer significantly influences the overall survival of elderly patients undergoing total laryngectomy for LC. Early diagnosis of the disease is crucial for patient survival.

## Introduction

Laryngeal cancer (LC) is one of the most common forms of head and neck cancer and may be a significant source of morbidity and mortality, particularly in the elderly population [1,2]. Worldwide, LC ranks 20th among all human neoplasia, 15th for men and 24th for women [3]. According to WHO, in 2022, there were 189,191 new cases of LC [4]. In Greece, a nation experiencing a demographic shift toward an aging population, each year, about 840 laryngeal cancers are diagnosed, with a higher proportion of males versus females [4-6].

LC can occur at any age, but the rate of new cases and deaths from laryngeal cancer is higher among people aged 65-74 [7]. Cancer stage at diagnosis determines treatment options and has a strong influence on the overall survival rate [7]. Moreover, the five-year survival rate ranges from 30% to 90% and also depends on age, general health and the location of the cancer (glottis, supraglottis, or subglottis) [8-11].

Throughout the last decade, the treatment approach is focusing on organ preservation with chemoradiation. However, in elderly patients or patients with poor general health, who may not tolerate the toxicity of chemoradiation, laryngectomy stands out as a cornerstone intervention, aiming to eradicate the malignancy and preserve the patient's overall quality of life [11,12]. Consequently, understanding the long-term outcomes and survival rates associated with laryngectomy in elderly patients remains a critical area of investigation. The aim of this study is to investigate the overall survival rate (five years) of elderly patients with LC who received total laryngectomy as the primary treatment and the impact of tumor-node-metastasis (TNM) staging on patients survival rates in the University Hospital of Patras, Western Greece.

## Materials and methods

Study design

A retrospective cohort study was conducted using data obtained from the Otorhinolaryngology Clinic of the University Hospital of Patras. The study included elderly patients aged 65 years and above who were diagnosed with laryngeal cancer and underwent total laryngectomy between the years 2000 and 2015. The tumors were staged using the 1997 American Joint Committee on Cancer (AJCC) and Union Internationale Contre le Cancer (UICC) criteria.

Patient selection

The study population consisted of elderly patients aged 65 years and above at the time of diagnosis with histologically confirmed laryngeal cancer, who underwent total laryngectomy as primary treatment. Patients with incomplete medical records or those who underwent partial laryngectomy were excluded from the study.

Data collection

Various demographic and clinical parameters were taken into consideration in the data collection process, including patient age, gender, smoking and alcohol use history, tumor histology, TNM staging, tumor location, treatment modalities (including total laryngectomy, neck dissection and adjuvant therapy) and survival outcomes. Patient records were meticulously reviewed to ensure accuracy and completeness of data.

Statistical analysis

The data gathered underwent analysis using IBM SPSS statistical software version V28 (IBM Corp, Armonk, NY, USA). Survival analysis was conducted using the Kaplan-Meier estimator to calculate overall survival rates, while the Log-rank test was utilized for intergroup comparisons. Subgroup analyses were performed to assess the impact of TNM staging and age on survival outcomes. Statistical significance was defined as the p-value being less than or equal to 0.05.

Ethics

This study was approved by the Ethics and Research Committee of the University Hospital of Patras (protocol number: 187/4-5-2023) and complies with all ethical standards for research.

## Results

During the period from 2000 to 2015, a total of 75 elderly patients aged 65 years and older underwent total laryngectomy. Among them, 73 patients (97.3%) were male, and two patients (2.7%) were female. The mean age of the patients was 73.88 years, with the majority falling within the age range of 65 to 75 years (n=46; 61.3%). Additionally, the vast majority of patients were smokers (n=72; 96%) and had a history of alcohol use (n=41; 54.7%) (Table 1).

**Table 1:** Demographic characteristics of study population Range denotes age range in years. n: number of participants; SD: standard deviation.

Characteristics	n	%	Mean ± SD	Range
Gender				
Male	73	97.3		
Female	2	2.7		
Age (year)			73.88 ± 6.63	65-89
65-75 years old	46	61.3		
>75 years old	29	38.7		
Smokers				
Yes	72	96		
No	3	4		
Alcohol use				
Yes	41	54.7		
No	34	45.3		
Total	75	100		

Of the total study population, 39 patients (52%) died during the observation period, while 36 patients (48%) remained alive at the end of the study. Among the deceased patients, the primary causes of death were attributed to distant metastasis (n=21; 53.8%), primary relapse and regional lymph node relapse (n=8; 20.5%) and other causes unrelated to the underlying disease (n=10; 25.6%) (Table 2). Specifically, the other causes unrelated to the underlying disease was as follows: two (20%) cardiac failure, two (20%) stroke, one (10%) pulmonary edema, one (10%) chronic renal insufficiency, one (10%) myelodysplastic syndrome and three (30%) unknown reason.

**Table 2:** Distribution of survival status and causes of death among deceased patients n: number of participants.

Characteristic	n	%
Survival		
Alive	36	48
Death	39	52
Total	75	100
Cause of death		
Metastasis	21	53.8
Relapse	8	20.5
Another cause	10	25.6
Total	39	100

According to the TNM system, the distribution of patients among different stages of LC revealed that eight patients (10.7%) were classified as stage II, 28 patients (37.3%) as stage III and 39 patients (52%) as stage IV. Regarding T (size or direct extent of the primary tumor) classification, 10 patients (13.3%) had T2 tumors, 26 patients (34.7%) had T3 tumors and 39 patients (52%) had T4 tumors, indicating a predominance of advanced-stage disease in the cohort study (Table 3).

**Table 3:** Histological and clinical characteristics of study population n: number of participants.

Characteristic	N	%
Stage		
II	8	10.7
III	28	37.3
IV	39	52
T: size or direct extent of the primary tumor		
Τ 2	10	13.3
Τ 3	26	34.7
Τ 4	39	52
N: degree of spread to regional lymph nodes		
Ν 0	70	93.3
Ν 1	4	5.3
Ν 2	1	1.3
M: presence of distant metastasis		
Μ 0	75	100
Tumor histology		
G1: well-differentiated	14	18.7
G2: moderately differentiated	49	65.3
G3: poorly differentiated	12	16
Location		
Supraglottis	26	34.7
Glottis	47	62.7
Subglottic	2	2.7
Surgery		
Total laryngectomy	23	30.7
Total laryngectomy and neck dissection	52	69.3
Adjuvant therapy		
No	47	62.7
Radiotherapy	18	24
Chemotherapy	1	1.3
Chemoradiotherapy	9	12
Total	75	100

The analysis of N (degree of spread to regional lymph nodes) classification showed that the majority of patients (n=70) had no regional lymph node involvement (N0, 93.3%), while a small proportion (n=5) had involvement of regional lymph nodes (N1, 5.3%; N2, 1.3%). Furthermore, all patients were classified as having no distant metastasis (M0, n=75; 100%) (Table 3).

Histologically, most of the tumors were moderately differentiated (G2, n=49; 65.3%), followed by poorly differentiated (G3, n=12; 16%) and well-differentiated (G1, n=14; 18.7%). The most common tumor locations were the glottis (n=47; 62.7%) and supraglottis (n=26; 34.7%), with a small proportion of cases occurring in the subglottic region (n=2; 2.7%) (Table 3).

**Figure 1:**
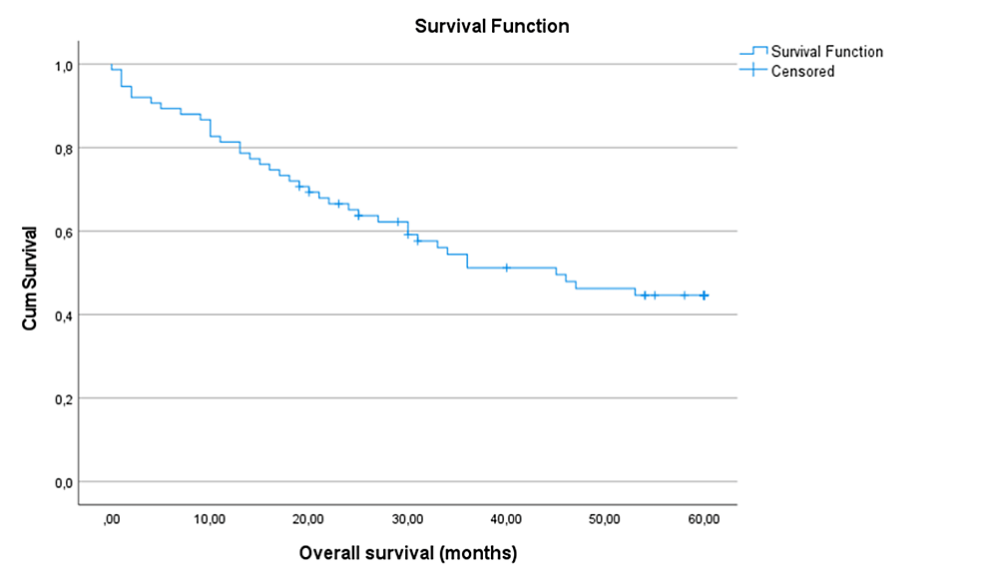
Five-year survival of the study population

In terms of treatment received, all patients underwent total laryngectomy, with 52 patients (69.3%) also undergoing neck dissection. Adjuvant therapy, including radiotherapy (n=18; 24%), chemotherapy (n=1; 1.3%) and chemo-radiotherapy (n=9; 12%), was administered to a subset of patients (Table 3). During follow-up period, five-year overall survival rate of the 75 patients with LC who received total laryngectomy was 44.6% (Figure 1).

**Figure 2:**
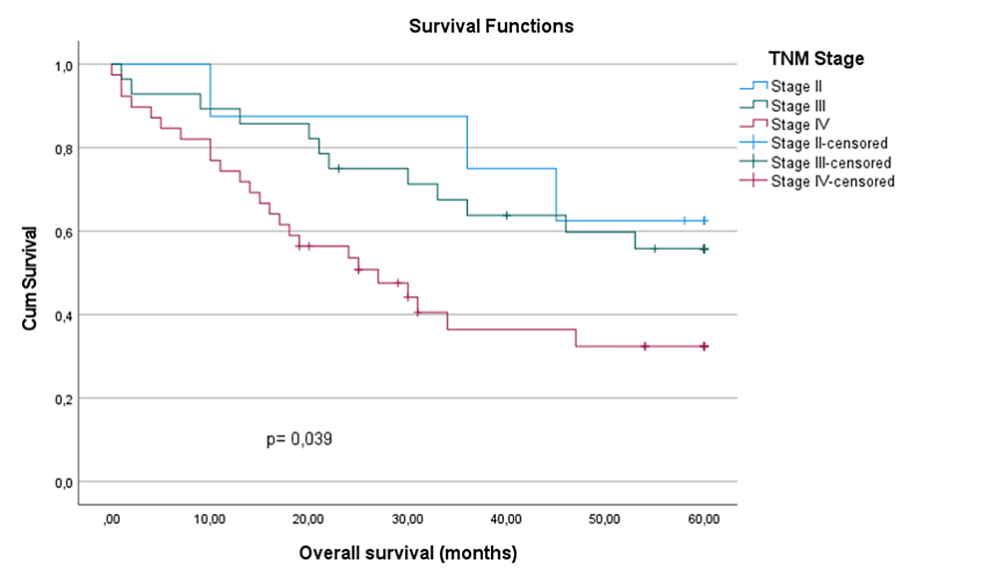
Kaplan-Meier survival curve by TNM staging TNM: tumor-node-metastasis.

However, the five-year overall survival rates varied significantly across different stages of LC. Specifically, the survival rates were 62.5% for stage II, 55.8% for stage III and 32.4% for stage IV (Figure 2). Statistical analysis using the Log-rank test revealed significant differences in survival rates among the TNM stages (χ²=6.474, p=0.039) (Table 4).

**Table 4:** Five-year survival rates and statistical significance by TNM staging, T, N and age p<0.05: Statistical significance level in comparison of groups; χ^2^: Chi‑square. TNM: tumor-node-metastasis; T: size or direct extent of the primary tumor; N: degree of spread to regional lymph nodes.

	5-year survival rate (%)	Log rank (x^2^)	p value
ΤΝΜ stage		6.474	0.039
II	62.5		
III	55.8		
IV	32.4		
T (ΤΝΜ)		6.382	0.041
T2	60		
T3	56.1		
T4	32.4		
N (ΤΝΜ)		19.135	<0.001
N0	47.1		
N1	-		
N2	-		
Age (year)		4.279	0.039
65-75 years old	51.7		
>75 years old	34.7		

Moreover, when considering T classification, notable differences in survival rates were observed. Patients with T2 tumors demonstrated a five-year survival rate of 60%, while those with T3 tumors exhibited a slightly lower rate of 56.1%. In contrast, patients with T4 tumors had the lowest five-year survival rate at 32.4%. The Log-rank test indicated a significant association between T classification and survival outcomes (χ²=6.382, p=0.041) (Table 4).

**Figure 3:**
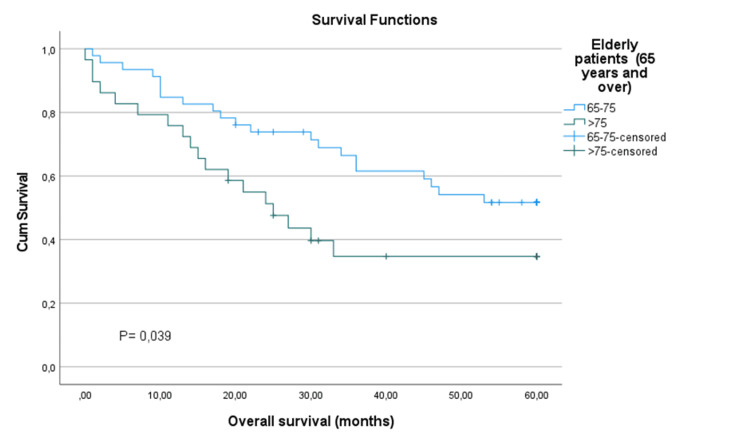
Kaplan-Meier survival curve by age group

Analysis of survival rates based on N classification revealed substantial variability. Patients classified as N0 had a five-year survival rate of 47.1%. However, due to the small sample sizes, statistical comparisons were not feasible for patients classified as N1 (n=4) or N2 (n=1) (Table 4). Regarding age, significant differences in survival rates were observed between different age groups. Patients aged 65-75 years exhibited a five-year survival rate of 51.7%, whereas those aged above 75 years had a notably lower rate of 34.7% (Figure 3). Statistical analysis demonstrated a significant association between age and survival outcomes (χ²=4.279, p=0.039) (Table 4).

## Discussion

In Greece, the number of elderly people over 65 is increasing and currently represents 23% of the total population [5]. Elderly patients is a population group which is typically affected by many comorbidities. As a result, these patients are especially vulnerable to loss of functional capacity, due to the interaction of their concurrent diseases [13-15]. LC is most often diagnosed in people aged 65 or above, with over 50% of the cases being new diagnoses [7].

Worldwide, the incidence of LC is higher in men than in women [16]. The prevalence of male patients in our study is consistent with existing literature, which may be due to biological markers or different lifestyles, such as tobacco and alcohol consumption [17,18]. Studies have highlighted a direct correlation between smoking and alcohol consumption at the appearance of LC [18,19]. In our study, most of the patients (96%) were smokers and 54.7% were alcohol users, evidence that supports the correlation between lifestyle considerations and the occurrence of LC.

According to literature data, the glottis is identified as the most common location for tumors, whereas occurrences in the subglottis are deemed extremely rare [20]. In our study, the distribution of tumor locations demonstrated a predilection for the glottis (62.7%) and supraglottis (34.7%), consistent with the anatomical sites commonly associated with laryngeal malignancies. In addition, a small percentage (2.7%) involved tumors in the subglottic region.

Histologically, the majority of tumors exhibited moderate differentiation (G2), comprising 65.3% of the cases. This histological profile suggests an intermediate level of tumor differentiation, characterized by varying degrees of cellular atypia and architectural disorganization. However, a notable proportion of tumors were classified as poorly differentiated (G3), representing 16% of the cases, indicating a more aggressive phenotype with a propensity for rapid growth and metastasis [21].

Based on the TNM staging system, a considerable proportion of patients were classified as stage III (37.3%) and stage IV (52%), indicating an advanced disease stage at the time of diagnosis. Further characterization based on T classification highlighted the prevalence of locally advanced tumors, with the majority of patients presenting with T3 (34.7%) and T4 (52%) tumors. This observation suggests extensive primary tumor involvement, which may pose challenges in surgical management and necessitate comprehensive treatment approaches to achieve optimal outcomes [12,22]. Similarly, the analysis of N classification demonstrated a substantial proportion of patients with no regional lymph node involvement (N0, 93.3%), indicating localized disease spread within the laryngeal region. However, a small but notable subset of patients exhibited regional lymph node metastasis (N1, 5.3%; N2, 1.3%).

Treatment modalities play a pivotal role in the management of LC. In our study, all patients underwent total laryngectomy, reflecting the standard surgical approach for locally advanced LC [23]. Moreover, a substantial proportion of patients (69.3%) underwent concurrent neck dissection, while adjuvant therapy (37.3%), including radiotherapy and chemotherapy, was administered to selected patients, based on individualized treatment protocols and disease-specific considerations. These findings underscore the multidisciplinary nature of LC care and the importance of tailored treatment strategies to address the unique needs of elderly patients [12,13].

Despite advancements in treatment modalities, the overall survival rates observed in our study do not differ from those reported in the international literature, particularly among patients with advanced-stage disease [11,24,25]. Specifically, patients with advanced-stage disease (stage IV) exhibited significantly lower five-year survival rates (32.4%) compared to those with early-stage disease (stage II, 62.5%). Similarly, patients with larger primary tumors (T4) demonstrated poorer survival outcomes (32.4%) compared to those with smaller tumors (T2, 60%). These findings underscore the importance of accurate staging in guiding treatment decisions and prognosticating outcomes for patients undergoing total laryngectomy.

Analysis of survival rates based on N classification revealed substantial variability, with patients classified as N0 demonstrating better survival outcomes compared to those with regional lymph node involvement. However, due to the small sample sizes, statistical comparisons were not feasible for patients classified as N1 or N2. Nonetheless, the presence of lymph node involvement remains an important prognostic factor that requires consideration in treatment planning.

Furthermore, advanced age has been identified as a significant predictor of poorer prognosis in various malignancies of the head and neck, including laryngeal cancer [22,26,27]. Our findings further support this association, as evidenced by the lower five-year overall survival rate observed among patients aged above 75 years (34.7%) compared to those aged 65-75 years (51.7%).

The retrospective study of elderly patients with laryngeal cancer who underwent total laryngectomy from 2000 to 2015 allowed us to understand the survival rates and the factors influencing it. Additionally, our findings underscore the need for continuous efforts to improve early detection, develop new therapeutic interventions and enhance supportive care measures to mitigate complications associated with treatment and improve the quality of life of elderly patients with laryngeal cancer in Western Greece.

However, this study has certain limitations that should be considered. First, the retrospective design may introduce bias. Second, the sample size of elderly patients in this single-center study may restrict the ability to detect small differences in survival. Third, the conclusion is based solely on univariate analysis. Finally, the study's focus on a specific geographic region (Western Greece) may limit the generalizability of the results to other populations.

## Conclusions

In conclusion, our study provides valuable insights into the demographic and clinical characteristics, as well as the prognostic factors (TNM staging and age) influencing survival outcomes among elderly patients with laryngeal cancer undergoing total laryngectomy. By considering TNM staging and age, healthcare professionals can better prognosticate outcomes and tailor treatment strategies to optimize survival and quality of life of elderly patients.
